# Suppression of acute proinflammatory cytokine and chemokine upregulation by post-injury administration of a novel small molecule improves long-term neurologic outcome in a mouse model of traumatic brain injury

**DOI:** 10.1186/1742-2094-5-28

**Published:** 2008-06-30

**Authors:** Eric Lloyd, Kathleen Somera-Molina, Linda J Van Eldik, D Martin Watterson, Mark S Wainwright

**Affiliations:** 1 Division of Critical Care, Department of Pediatrics, Children's Memorial Hospital, 2300 Children's Plaza, Chicago, IL 60614, USA; 2 Division of Neurology, Department of Pediatrics, Children's Memorial Hospital, 2300 Children's Plaza, Chicago, IL 60614, USA; 3 Center for Drug Discovery and Chemical Biology, Northwestern University, 303 E. Chicago Ave, Mailcode W896, Chicago, IL 60611, USA; 4 Center for Interdisciplinary Research in Pediatric Critical Illness and Injury, Children's Memorial Research Center, 2430 N. Halstead, Chicago, IL 60614, USA

## Abstract

**Background:**

Traumatic brain injury (TBI) with its associated morbidity is a major area of unmet medical need that lacks effective therapies. TBI initiates a neuroinflammatory cascade characterized by activation of astrocytes and microglia, and increased production of immune mediators including proinflammatory cytokines and chemokines. This inflammatory response contributes both to the acute pathologic processes following TBI including cerebral edema, in addition to longer-term neuronal damage and cognitive impairment. However, activated glia also play a neuroprotective and reparative role in recovery from injury. Thus, potential therapeutic strategies targeting the neuroinflammatory cascade must use careful dosing considerations, such as amount of drug and timing of administration post injury, in order not to interfere with the reparative contribution of activated glia.

**Methods:**

We tested the hypothesis that attenuation of the acute increase in proinflammatory cytokines and chemokines following TBI would decrease neurologic injury and improve functional neurologic outcome. We used the small molecule experimental therapeutic, Minozac (Mzc), to suppress TBI-induced up-regulation of glial activation and proinflammatory cytokines back towards basal levels. Mzc was administered in a clinically relevant time window post-injury in a murine closed-skull, cortical impact model of TBI. Mzc effects on the acute increase in brain cytokine and chemokine levels were measured as well as the effect on neuronal injury and neurobehavioral function.

**Results:**

Administration of Mzc (5 mg/kg) at 3 h and 9 h post-TBI attenuates the acute increase in proinflammatory cytokine and chemokine levels, reduces astrocyte activation, and the longer term neurologic injury, and neurobehavioral deficits measured by Y maze performance over a 28-day recovery period. Mzc-treated animals also have no significant increase in brain water content (edema), a major cause of the neurologic morbidity associated with TBI.

**Conclusion:**

These results support the hypothesis that proinflammatory cytokines contribute to a glial activation cycle that produces neuronal dysfunction or injury following TBI. The improvement in long-term functional neurologic outcome following suppression of cytokine upregulation in a clinically relevant therapeutic window indicates that selective targeting of neuroinflammation may lead to novel therapies for the major neurologic morbidities resulting from head injury, and indicates the potential of Mzc as a future therapeutic for TBI.

## Background

Traumatic brain injury (TBI) is a leading cause of death in Western industrialized nations [1,2], with an estimated 50,000 deaths annually in the United States [3]. The causes of TBI vary with age but the medical and financial impact of these injuries is substantial [4,5]. In the United States alone, an estimated 1.6 million cases of TBI occur annually, with approximately 300,000 cases of sufficient severity to require hospitalization [6,7]. The mortality with severe TBI can reach 40% and neurologic morbidity among survivors is high [8,9]. The neurologic sequelae in survivors of TBI include cognitive impairment, dementia, epilepsy, depression and neurodegenerative disease [10]. Current standards of care for TBI focus largely on supportive measures [11]. There is a major unmet need for TBI therapies that attenuate long-term, functional neurologic deficits [12-14].

Insults to the central nervous system (CNS) induce a neuroinflammatory response characterized by activation of microglia and astrocytes, damage to the blood-brain-barrier (BBB), and acute up-regulation of proinflammatory cytokines such as interleukin (IL)-1β, tumor necrosis factor (TNF)α, and IL-6. In the case of TBI, this complex neuroinflammatory cascade can lead to opposing effects [15]: beneficial outcomes through production of reparative and protective factors, or detrimental outcomes when the production of proinflammatory mediators is prolonged, excessive, or temporally inappropriate [for review, see [16]]. There is increasing recognition that suppression of the CNS proinflammatory cytokine cascade should be explored as a therapeutic approach to TBI because of its contribution to secondary injury that includes cerebral edema, neuronal damage and cytotoxicity.

A variety of studies using pharmacological or genetic methods have demonstrated beneficial effects of suppressing the CNS proinflammatory cytokine cascade induced by TBI [17-22]. For example, treatment of rats with IL-1 receptor antagonist (IL-1ra), a protein that antagonizes IL-1 activity, administered either by intracerebroventricular administration [23], or by implantation of IL-1ra-expressing fibroblasts into the wound cavity [22] reduced the extent of neurologic injury after experimental head injury. Similar protection was found in transgenic mice with CNS-selective over-expression of IL1ra [24]. Other studies showed that suppression of TNFα production or activity by administration of small molecules (HU-211, pentoxifylline) or a TNFα binding protein reduced neurologic injury [25-27].

Taken together, preclinical data indicate that targeting glia proinflammatory cytokine overproduction may represent an effective new therapeutic intervention for TBI. However, many current cytokine-modulating drugs are macromolecules, and using macromolecules as a therapeutic approach has a number of disadvantages, such as instability, high cost and potential for immune responses to the therapy. There is an unmet clinical need for a small molecule therapeutic that attenuates the acute cytokine and chemokine surge with resultant improvement in longer term neurologic outcomes when the drug is administered in a clinically relevant time window following the injury [28].

In the present studies, we tested the hypothesis that suppression of the acute increase in proinflammatory cytokines following TBI would attenuate neurologic injury and neurobehavioral impairment. As a step toward addressing the need for novel therapeutics, we explored the potential utility of Minozac (Mzc) administered hours post-injury in a murine closed-skull, cortical impact model of TBI. Mzc [29] is a bioavailable, brain-penetrant, small molecule experimental therapeutic that improves synaptic dysfunction and neurobehavioral impairment when administered after the initiation of the injury stimulus in animal models of epilepsy [30] and Alzheimer's disease [29]. The mechanism of Mzc action is selective reduction of excessive proinflammatory cytokine production by activated glia back towards basal levels. We report here that administration of Mzc at 3 h and 9 h following TBI attenuates the acute increase in proinflammatory cytokine and chemokine levels and reduces the longer term astrocyte activation, neurologic injury and neurobehavioral deficits observed over a 28-day recovery period. Mzc-treated animals also have no significant increase in brain water content (edema), a major cause of the neurologic morbidity associated with clinical TBI [11]. These data lend support both to the potential of glial activation as a therapeutic target in acute brain injury and the utility of Mzc for the treatment of TBI.

## Methods

### Animal care and housing

All experiments were performed in accordance with the National Institutes of Health Guide for Care and Use of Laboratory Animals. All experimental procedures were approved by Children's Memorial Research Center Institutional Animal Care and Use Committee. Adult male CD-1 mice weighing between 20–30 gm were used for these experiments.

### Mouse model of closed head injury

Mice were subjected to closed head injury using a stereotactically guided pneumatic compression device with minor modification of published methods [20,31-33]. Mice were anesthetized with isoflurane (4% induction, 1.5% maintenance) in 100% oxygen. Endotracheal intubation was performed using an otoscope as a laryngoscope and 18 gauge angiocatheter as an endotracheal tube. Mice were mechanically ventilated (Hugo Sachs Electronik, March-Hugstetten, Germany), using a protective ventilation strategy (3 cm H_2_O positive end-expiratory pressure; tidal volume 5 cc/kg) as previously described [34,35]. Core temperature was monitored using a rectal probe (IT-18 Physitemp, NJ) and maintained at 37.0 ± 0.1°C by surface heating and cooling. Mice were secured in prone position in a customized resin mold, the scalp shaved and prepared with betadine. A midline sagittal scalp incision was made using sterile technique, and the periosteum reflected to reveal the appropriate landmarks. A concave 3 mm metallic disk was affixed in the midline, immediately caudal to Bregma. A single controlled midline skull impact was delivered using a pneumatic impactor (Air-Power Inc., High Point, NC) using a 2.0 mm steel tip impounder at a controlled velocity (6.0 ± 0.2 m/s) and impact depth (3.0 mm). Mice with depressed skull fracture or visible hemorrhage were excluded from the study. After impact, the scalp incision was sutured, and mice were allowed to achieve spontaneous respiratory effort prior to extubation. Sham-injured animals underwent identical surgical procedures as the trauma group, but no impact was delivered.

### Minozac production and treatment protocol

Mzc was synthesized by the production scheme previously described [29], dissolved in sterile saline, and administered to mice by intraperitoneal (i.p.) injection at 3 h and 9 h after TBI (5 mg/kg Mzc per dose). Controls were TBI-treated or sham-treated mice administered an equivalent volume of saline vehicle.

### Collection of brain tissue

At selected survival times, mice were anesthetized under isoflurane, sacrificed and perfused via the left ventricle with 15 ml of chilled phosphate buffered saline (PBS), followed by a second 15 ml perfusion with either PBS (Western blotting and ELISA) or 4% paraformaldehyde in PBS (immunohistochemistry). For immunohistochemistry, the brains were manually dissected from the calvarium with both cerebral hemispheres intact minus the cerebellum, and immersed in 4% paraformadelyde overnight at 4°C before embedding in paraffin.

### Brain extract preparation and determination of protein concentration

For recovery up to 12 hours, hippocampal and cortex extracts were prepared from both hemispheres by sonication in protease inhibitor cocktail comprising 1 μg leupeptin (Sigma, St. Louis, MO), 0.001 M 4-dithio-L-threitol (DTT, Sigma), 0.002 M sodium orthovanadate (Sigma) and 0.001 M phenylmethanesulfonyl fluoride (PMSF, FLUKA, Switzerland) in 1 ml PBS as previously described [30]. The impact zone was defined as a region 0.5 mm anterior to Bregma and extending 3 mm caudal to Bregma, encompassing the region covered by the diameter (3 mm) of the disk affixed to the skull. The extracted region comprised the cortex covered by the disk in addition to the hippocampi. Briefly, tissue was centrifuged at 4°C for 10 minutes, the supernatant collected and total protein concentration was measured in the supernatant using commercially available reagents (BCA, Pierce, Rockford, IL).

### Measurement of proinflammatory cytokines and chemokines

Levels of IL-1β, IL-6, TNF-α and CCL2 were measured in hippocampal and cortical supernatants by sandwich immunoassay methods using commercially available electrochemiluminescent detection system, plates, and reagents (Meso-Scale Discovery (MSD), Gaithersburg, Maryland) [30]. For each assay, samples were analyzed in duplicate and compared with known concentrations of protein standard. Plates were analyzed using the SECTOR Imager 2400.

### Immunohistochemistry

Immunohistochemical detection of hippocampal neuronal injury was performed in 5 μm paraffin-embedded sections using Vectastain Elite ABC immunodetection kits and diaminobenzidine substrate (DAB) (Vector Laboratories, Burlingame CA). Astrocyte activation was measured using an antibody to the glial-derived protein S100B (1:1500, rabbit polyclonal, DAKO Cytomation, Carpinteria, CA). Neuronal injury was assessed using the neuronal nuclei marker NeuN (mouse monoclonal, 1:25, Chemicon International, Temecula, CA). Control sections were incubated in normal serum or PBS in place of primary antibody. In order to determine the specificity of the antibodies, selected slides were incubated with preadsorbed IgG in place of primary antibody. Sections were incubated with primary antibody or controls overnight in a sealed humidity chamber at 4°C, washed, and then incubated for one hour at 38°C with the appropriate biotinylated secondary antibody at 1:400 dilution (Vector).

### Image acquisition and quantification of immunoreactive cells

For each animal, 12 non-consecutive sections were immunostained, representing the extent of the hippocampus (approximately Bregma 0.50 mm to Bregma -3.0) and quantified. Hippocampal sections were examined under brightfield microscopy by two blinded observers (Nikon Eclipse E800). Regions CA1, CA2, CA3, dentate gyrus (DG) and polymorph dentate gyrus (PoDG) of the hippocampus were photographed at 20× magnification. For S100B, digitized images were converted to grayscale for quantification of immunoreactive cells. The percentage of positive cells in the hippocampal regions was measured by thresholding for dark objects indicative of immunoreactive cells (Metamorph, Universal Imaging Corporation, Sunnyvale, CA). The total hippocampal immunoreactivity was obtained for each sample as previously described [30]. Neuronal injury in NeuN stained sections was scored by region (CA1-3, DG, PoDG) in the hippocampus for each section by a blinded observer. Each region was assigned a score as 1 (normal), 2 (moderate injury) and 3 (severe injury) based on the morphologic appearance of neurons. The criteria for determining the presence of neuronal injury were the presence of areas of condensed or pale neurons. The injury was defined as moderate if present in only one region examined and severe if present in more than one region.

### Hippocampal-linked task behavioral testing

The Y-maze test of spontaneous alternation was used to evaluate hippocampus-dependent spatial learning [30,36]. Testing began on recovery day 7 to diminish the effects of motor impairment produced by TBI [31]. Testing was performed by a blinded observer daily until day 28 of recovery. Each animal started in the vertical arm of the Y-maze. If the animal selected a different arm on the second run in the maze, it was scored as alternating. The percent alternation over the duration of testing was calculated for each animal.

### Wet-to-dry method of assessing brain water content

To quantify cerebral edema following TBI, brain water content was measured using published methods [32,37]. Briefly, brains were removed 24 hours after TBI or sham procedure and cerebellum and hindbrain removed. Using a mouse brain slicer (Harvard Apparatus) a 6 mm coronal section was dissected from the impact site and immediately weighed. To determine water content, samples were placed on aluminum foil, dried at 105°C for 48 hr and reweighed. Brain water content was calculated as the difference in weights between wet and dry weight.

### Statistical analysis

Values are expressed as mean ± SEM for each group. For neuronal injury, data are expressed as median score ± IQR. Test for normality was performed for each data set. For comparisons of three or more groups, One-way analysis of variance (ANOVA) was performed, followed by Tukey's Multiple Comparison Test. Two groups were compared using Student's t-test. Significance was defined as p < 0.05. Prism 4.0 (GraphPad Software, Inc., San Diego, CA) was used for statistical analyses.

## Results

**Figure 1:**
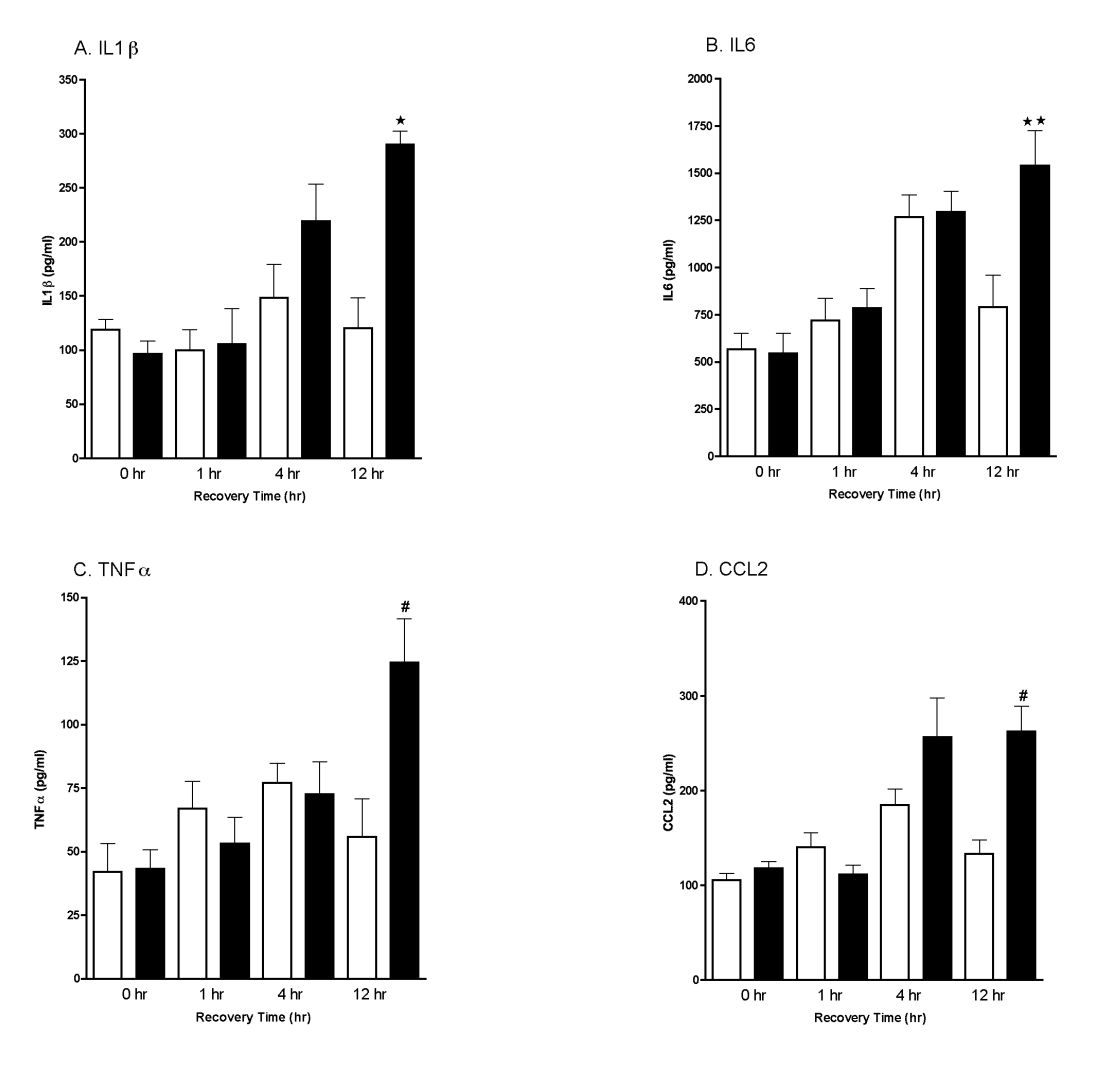
**Time course of acute changes in proinflammatory cytokines following TBI**. Levels of the cytokines IL-1β (A), IL6 (B), TNFα (C) and the chemokine CCL2 (D) in pooled hippocampus and cortex extracts following sham procedure (open bars) or closed head TBI (filled bars) were measured by ELISA. Animals were sacrificed at 0-, 1-, 4-, and 12-hr recovery. Data are expressed as mean ± S.E.M of n = 6–8 animals per group. Significantly different from sham: *P < 0.05 *vs *Sham control; **P < 0.01 *vs *Sham; ^#^P < 0.001 *vs *Sham by ANOVA.

**Figure 2:**
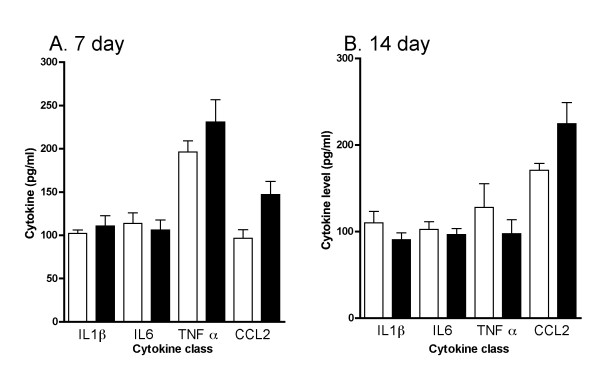
**Time course of long-term changes in proinflammatory cytokines following TBI**. Levels of the cytokines IL-1β, IL6, TNFα, and the chemokine CCL2 in pooled hippocampus and cortex extracts following sham procedure (open bars) or closed head TBI (filled bars) were measured by ELISA. Animals were sacrificed at 7 (A) and 14 (B) day recovery. Data are expressed as mean ± S.E.M of n = 6–8 animals per group. There were no significant differences between groups at either time point.

Mice were subjected to a closed-skull cortical impact (TBI) or sham procedure, and the time course (Fig. 1) of the acute increase in levels of proinflammatory cytokines (IL-1β, IL6 and TNF-α) and the chemokine CCL2 was determined. Levels of the cytokines/chemokine in pooled hippocampus and cortex extracts were measured at 0-, 1-, 4-, and 12-hr after TBI or sham procedure (n = 4 per group). There was a trend toward an increase at 4-hr post injury but this did not reach significance. By 12 hours after injury, levels of IL-1β, IL6, TNF-α, and CCL2 were significantly increased compared to sham controls. To confirm that the cytokine and chemokine response to injury was transient, we measured cytokine levels at 7 and 14 days after TBI or sham procedure (Fig. 2). There were no differences between Sham and TBI groups at either of these later timepoints for any of the cytokines examined.

**Figure 3:**
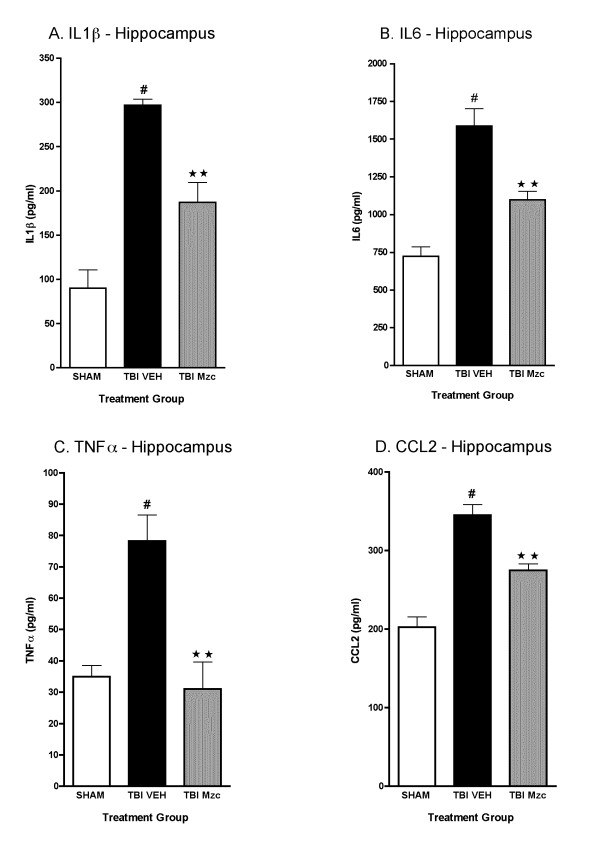
**Minozac suppresses proinflammatory cytokine upregulation in hippocampus following TBI**. Mice were subjected to TBI or sham procedure. At 3 hr and 9 hr following TBI, mice were injected with Mzc (5 mg/kg/dose) or saline diluent (VEH). Mice were sacrificed at 12 hr post-injury, and levels of the proinflammatory cytokines IL-1β (A), IL6 (B), TNFα (C) and the chemokine CCL2 (D) in hippocampal extracts were measured by ELISA. Mzc treatment resulted in significant attenuation of the increase in cytokines measured in the TBI group treated with saline vehicle. Cytokine levels in the Mzc-treated TBI mice were not significantly different from the sham controls. Data are expressed as mean ± S.E.M of n = 5–7 animals per group. ^#^P < 0.001 *vs *Sham control; **P < 0.01 *vs *TBI-VEH by ANOVA.

**Figure 4:**
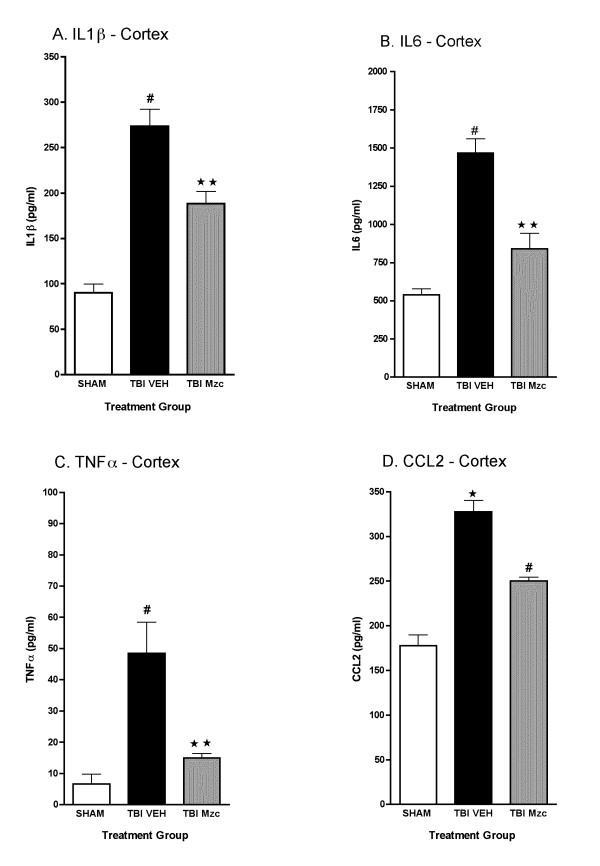
**Minozac suppresses proinflammatory cytokine upregulation in cortex following TBI**. Mice were subjected to TBI or sham procedure. At 3 hr and 9 hr following TBI, mice were injected with Mzc (5 mg/kg/dose) or saline diluent (VEH). Mice were sacrificed at 12 hr post-injury, and levels of the proinflammatory cytokines IL-1β (A), IL6 (B), TNFα (C) and the chemokine CCL2 (D) in cortical extracts were measured by ELISA. Mzc treatment resulted in significant attenuation of the increase in cytokines measured in the TBI group treated with saline vehicle. Cytokine levels in the Mzc-treated TBI mice were not significantly different from the sham controls. Data are expressed as mean ± S.E.M of n = 5–7 animals per group. ^#^P < 0.001 *vs *Sham control; **P < 0.01 *vs *TBI-VEH by ANOVA.

We used Mzc, a brain-penetrant, small molecule inhibitor of proinflammatory cytokine upregulation [29,30] to attenuate the increase in proinflammatory cytokines after TBI. We selected a time window of treatment based on two major considerations. First, the results of the previous experiments showed increases in cytokines levels by 12 hours after TBI, reflecting an earlier increase in cytokine biosynthesis, processing and release. Second, a realistic clinical therapeutic window for time from injury to trauma center would be approximately three hours or less. Mice were treated with Mzc or diluent (Saline) at 3 and 9 hr after TBI. Cytokine levels were measured in hippocampal and cortical extracts at 12 hr following TBI or sham procedure. In hippocampal extracts (Fig. 3) of injured animals, those treated with Mzc showed a significant reduction in IL-1β (A), IL6 (C), TNF-α (C), and CCL2 (D) compared to saline-treated animals. Indeed, Mzc treatment of injured animals reduced cytokine levels such that they were not significantly different from sham-injured animals. A similar pattern was present in cortex (Fig. 4), with Mzc treatment reducing the cytokine levels back toward basal.

**Figure 5:**
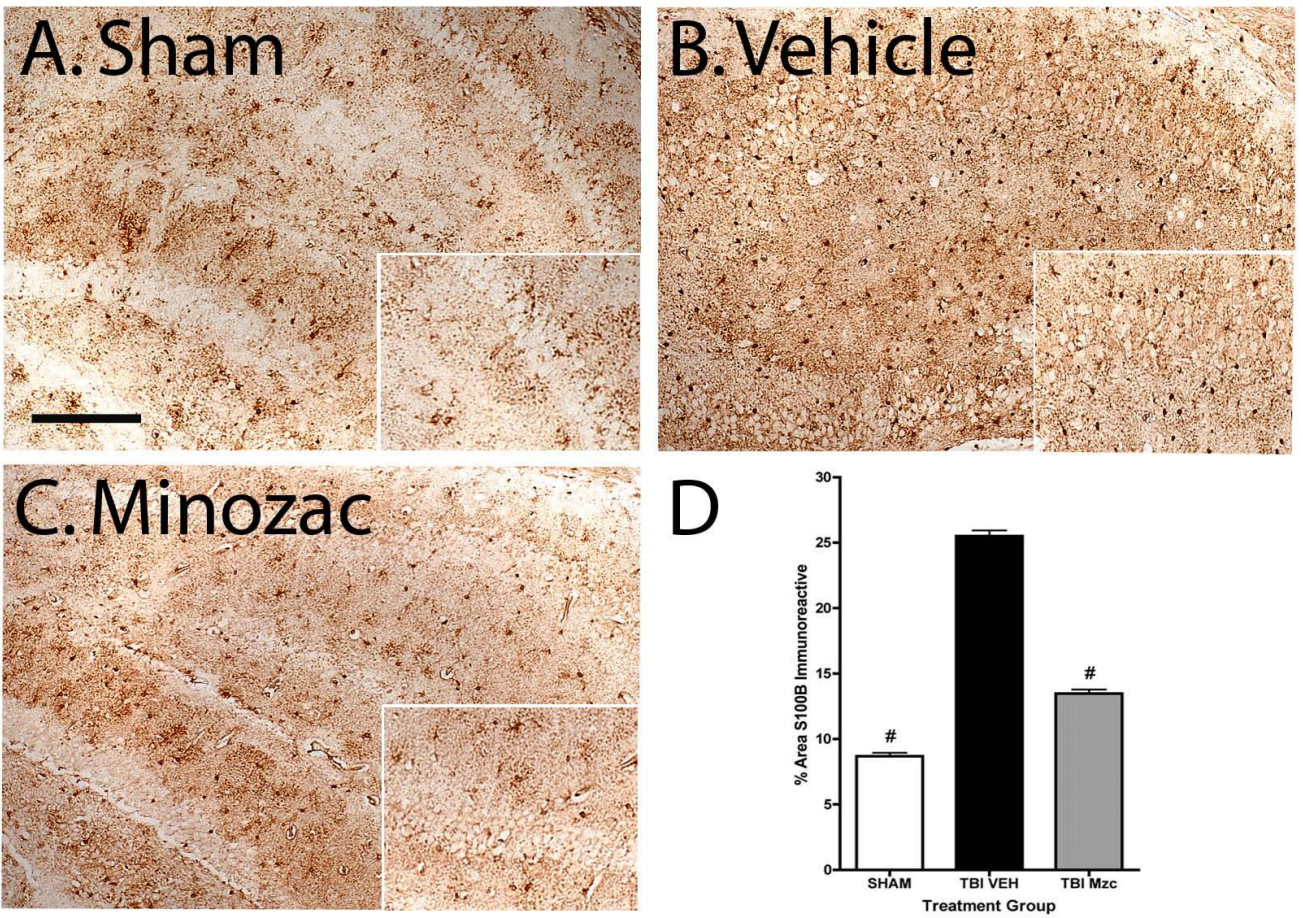
**Quantification of S100B immunoreactive cells after 28 day recovery following TBI**. Mice were subjected to TBI or sham procedure. At 3 hr and 9 hr following TBI, mice were injected with Mzc (5 mg/kg/dose) or saline diluent (VEH). After 28 day recovery, S100B immunoreactive cells in the hippocampus were quantified in sham controls (A), TBI treated with saline vehicle (B) and TBI treated with Mzc (C). Representative sections show an increase in the injured animals (B) which was prevented by treatment with Mzc (C). Insets (A-C) show high power image of hippocampal neuronal layer. Quantification of the digitized images (D) shows a reduction in astrocyte activation in the Mzc-treated group. Data are expressed as mean ± S.E.M of n = 5–7 animals per group. ^#^P < 0.001 *vs *TBI-Veh by ANOVA. Bar = 100 μm.

To determine whether glia remained activated following TBI, we used immunohistochemical methods to measures changes in the expression of the astrocyte protein S100B over 28 day recovery after TBI or sham procedure (Fig. 5). To determine whether attenuation of the acute increase in proinflammatory cytokines prevented long-term astrocyte activation, we treated mice with Mzc under the same conditions as the previous experiment. Following TBI, there was a significant increase in the hippocampus in the number of S100B immunoreactive cells after 28 day recovery compared to sham controls. In the Mzc-treated group following TBI, this increase was prevented.

**Figure 6:**
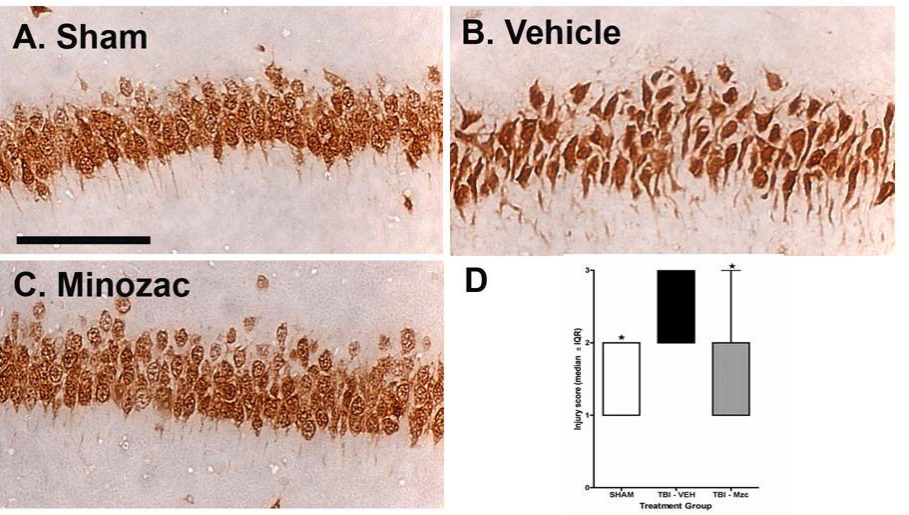
**Minozac attenuates neuronal injury after 28 day recovery following TBI**. Mice were subjected to TBI or sham procedure. At 3 hr and 9 hr following TBI, mice were injected with Mzc (5 mg/kg/dose) or saline diluent (VEH). After 28-day recovery, neuronal injury was quantified in each region of the hippocampus based on the morphologic appearance of the NeuN-labeled neurons and a score (1 = normal; 2= moderate injury; 3= severe injury) assigned for each region. Representative sections are shown for sham controls (A), TBI treated with saline vehicle (B) and TBI treated with Mzc (C). Neurons in injured animals treated with saline were shrunken and dystrophic (B) compared to both the injured animals treated with Mzc (C) and sham controls (A). (D) Quantification of the regional injury scores between groups. Data are expressed as median injury score ± IQR of n = 5–7 animals per group. *P < 0.05 vs TBI-VEH by ANOVA. Bar = 100 μm.

To determine whether the effects of Mzc on suppression of the acute TBI-induced cytokine increases resulted in reduction in longer-term neurologic injury, we used immunohistochemical methods to measure changes in the expression of the neuronal protein NeuN (Fig. 6). The morphology of the NeuN-labeled cells in the TBI group treated with vehicle (Fig. 6B) was highly abnormal, manifesting small and dystrophic-appearing cell bodies, compared to sham controls (Fig. 6A). In contrast, the majority of neurons in the hippocampi of mice subjected to TBI and treated with Mzc appeared normal (Fig. 6C). Analysis of hippocampal sections (Fig. 6D) showed a significant increase in neuronal injury in the mice subjected to TBI compared to saline controls, and the injury was prevented by Mzc treatment. These results demonstrate that Mzc treatment at 3 and 9 hr after TBI reduces the neuronal damage seen at 28 days after injury.

**Figure 7:**
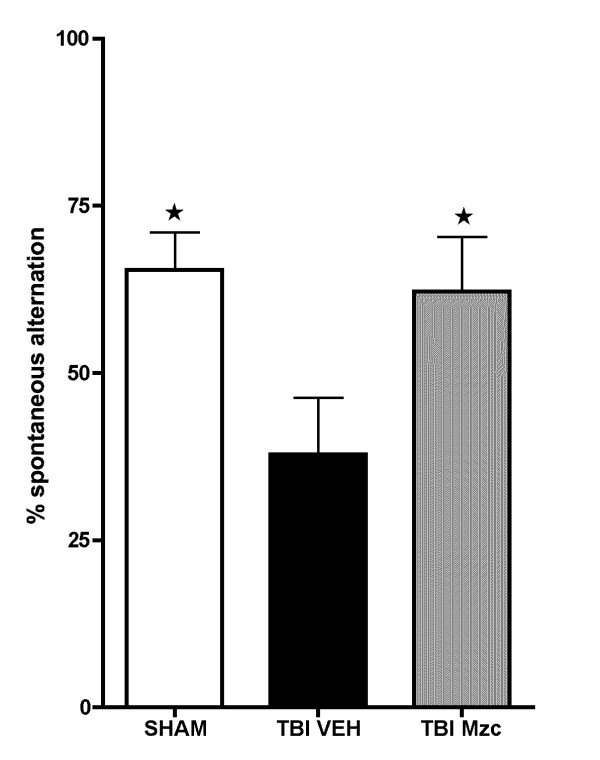
**Minozac attenuates hippocampal-dependent Y-maze behavioral impairment following TBI**. Mice were subjected to TBI or sham procedure. At 3 hr and 9 hr following TBI, mice were injected with Mzc (5 mg/kg/dose) or saline diluent (VEH) via intraperitoneal injection. On days 7 through 28 of recovery, hippocampal function was assessed by alternation in the Y-maze. Treatment with Mzc prevented neurobehavioral impairment resulting from TBI. Data are expressed as mean ± S.E.M of n = 5–7 animals per group. *P < 0.05 *vs *TBI-VEH by ANOVA.

To determine whether the protection against hippocampal neuropathologic injury afforded by Mzc resulted in improvement in behavioral function, we measured performance in the Y-maze (Fig. 7), a test of hippocampal-linked behavior [29,30]. We performed daily testing for alternation over days 7 to 28 of recovery after TBI or sham operation. Following TBI, there was a significant reduction in Y-maze performance compared to sham controls. However, treatment with Mzc following TBI prevented the Y-maze behavioral deficit.

**Figure 8:**
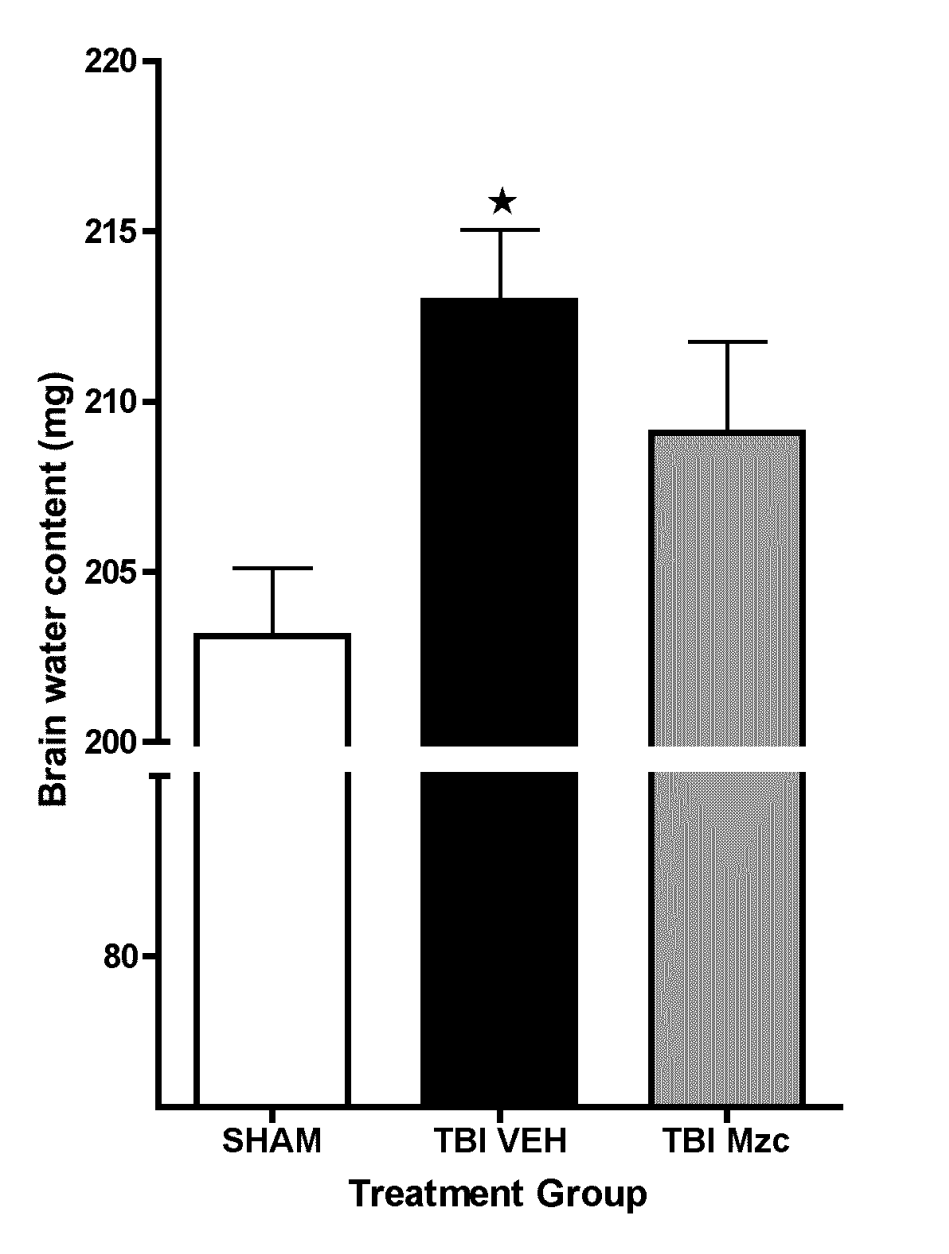
**Minozac treated animals have attenuated increase in brain water content following TBI**. Mice were subjected to TBI or sham procedure. At 3 hr and 9 hr following TBI, mice were injected with Mzc (5 mg/kg/dose) or saline diluent (VEH) via intraperitoneal injection. Mice were sacrificed 24 hr post-injury, and brain water content was measured in coronal sections by wet dry methods. Water content increased significantly following TBI in mice treated with saline but not when treated with Mzc. Data are expressed as mean ± S.E.M of n = 9–13 animals per group. *P < 0.05 *vs *sham control by ANOVA.

The protection afforded by treatment with Mzc occurred during the period after TBI in which cerebral edema is starting to evolve, which is a major cause of the neurologic morbidity associated with TBI [11]. To determine whether there also was a reduction in cerebral edema over this period, we measured brain water content after 24 hr after TBI (Fig. 8). There was a significant increase in water content (data expressed as mg ± SEM; n) following TBI (213.0 ± 2.1; 13) compared to sham controls (203.1 ± 2.0; 9)(p < 0.05 by ANOVA). Brain water content in animals treated with Mzc after TBI (209.1 ± 2.7; 13) was not significantly different from the sham group.

## Discussion

The key findings of this study are that delayed administration of a small molecule inhibitor of proinflammatory cytokine upregulation, Mzc, attenuates the acute increase of brain proinflammatory cytokines and chemokines after TBI in a mouse model and that this suppression results in a longer term reduction in neuropathologic injury and neurobehavioral impairment. These findings, and the efficacy of Mzc in a clinically relevant therapeutic window (3 h and 9 h post-injury), are consistent with glial activation as a therapeutic target in both acute [30] and chronic [29,38,39,56,62] neurologic injury models.

The astrocyte-derived protein S100B is increased after TBI [16] as was found in this study by immunohistochemical staining, demonstrating a persistent glial activation after the acute insult. S100B is a pleiotropic protein that exhibits neurotrophic and neuroprotective activities at low nanomolar concentrations, but causes excessive glial activation and neuronal injury at high nanomolar to low micromolar concentrations [63,66]. Clinical and pre-clinical studies have suggested that elevated S100B levels in serum or CSF are inversely related to outcome after TBI while other studies have identified a restorative role for S100B after TBI (for review, see 16). The attenuation of the increase in S100B immunoreactivity in the Mzc-treated animals indicates a potential role for activated glia in the mechanisms of neuronal dysfunction after TBI. This is consistent with our previous findings in a rodent model of early-life seizures [30] in which suppression of early cytokine elevation after seizures prevented long-term increases in S100B and was associated with improved neurobehavioral function.

Current standards of care for TBI are primarily supportive and focus on the reduction of secondary injury [11,38]. Therapies which prevent the long-term neurologic sequelae of TBI, including cognitive impairment [39,40] and the resulting decline in psychosocial functioning [9,41], are currently lacking. Recently, an apoE mimetic peptide has been shown to reduce microglial activation and neuronal death, and improve short-term sensorimotor function when administered two hours after TBI [42]. In the present study, the small molecule, Mzc, restored mouse brain proinflammatory cytokine levels back towards normal and resulted in later improvement in synaptic dysfunction and behavioral outcomes. Taken together, these results add to the preclinical evidence in support of targeting the upregulation of proinflammatory cytokines and chemokines as a therapeutic approach in TBI, and extend the therapeutic window for such intervention.

A secondary finding of this study is the small, but potentially clinically significant, lessening of TBI-induced increase in brain water content in the Mzc-treated animals. Neuroinflammation has been implicated as a contributor to cerebral edema, a major factor in the neurologic morbidity associated with TBI [11]. Although the mechanisms of such blood-brain-barrier (BBB) dysfunction are not fully elucidated, the chemokine CCL2 [43] and the proinflammatory cytokine TNFα modulate BBB function via regulation of angiotensin II [44]. Our finding that Mzc treatment attenuates the TBI-induced increases in both CCL2 and TNFα raises one of several possible mechanisms contributing to this aspect of the clinical presentation.

The rapid increase in proinflammatory cytokines and chemokines after TBI in the mouse closed head injury model described here is consistent with previous brain injury studies and congruent with clinical findings. For example, TNF increases within hours of trauma [45], and clinical studies of TBI patients show increases of TNF levels in serum and cerebrospinal fluid [46]. Elevated levels of the chemokine CCL2 occur in response to mechanical [47,48] and other forms [49] of brain injury. This increase occurs within hours after the insult [50]. Similarly, the IL-1 family of cytokines increase within hours [51] after brain injury and may act synergistically with TNF to increase injury [52]. It should be noted that the sham procedure itself, as expected, does cause some increases in cytokine levels that vary with the cytokine measured. For example, the levels of IL6 and TNFα in the sham controls at the early time points may reflect an acute response to the sham procedure. However, only TNFα levels in the sham controls remain increased at the later time points, with other cytokine and chemokine levels measured by a multiplex procedure from the same biological specimen returning toward basal levels. The reason for the higher levels in shams for this single cytokine is not known. Regardless, these results do not alter the significance of the findings reported here as there is a clear increase above sham controls in the animals receiving the calibrated brain injury.

A causal linkage between changes in proinflammatory cytokine levels and neurologic outcomes is indicated by the improvement in neurologic endpoints after suppression of proinflammatory cytokine levels back towards control by Mzc treatment. This is consistent with previous reports demonstrating the potential for targeting selective aspects of glial activation in a variety of injury models [29,30,53-56]. The small molecule used in this report, Mzc, is a bioavailable, water-soluble, CNS-penetrant, compound that restores hippocampal pro-inflammatory cytokine up-regulation back towards basal when administered at comparatively low doses. Previous studies of post-injury treatment with Mzc in other rodent injury models, including kainic acid-induced seizures [30] and human Abeta-induced neuronal toxicity [29], have shown that the restoration of the proinflammatory cytokine increases back towards basal is linked to a reduction in long-term neuronal injury and attenuation of hippocampal-dependent behavioral deficits. Here, we extend the evidence identifying proinflammatory cytokine modulation as a therapeutic approach to TBI by showing that the treatment window can be extended to three hours for an acute closed-head injury, and showing sustained preservation of hippocampal-dependent function over an extended recovery period well after cessation of Mzc administration. Although the results reported here provide a precedent for altering neurologic outcomes observed well after Mzc intervention, it will be important in future studies to examine longer-term recovery for periods greater than one month.

Major determinants of efficacy, lack of efficacy, or toxicity in a therapeutic intervention study include the therapeutic compound's properties, the dose administered, the timing of therapeutic intervention, the particular form of injury, and the therapeutic target. These are critical considerations for neuroinflammatory responses to CNS injury as these processes can be both beneficial and injurious. In the case of TBI, the neuroinflammatory response can potentially be reparative and neuroprotective [15,57,58]. A relevant example for the clinical trial of immune modulation for treatment of TBI is the CRASH study [58] in which the efficacy of steroid treatment was examined. The relative risk of dying from all causes in the first 2 weeks after TBI was increased by treatment with steroids compared to the placebo-treated group. However, steroids used as drugs are well known for their diverse and sometimes divergent outcomes that can vary with the type of injury as well as drug dosing [59,60]. Similarly, chronic dosing over a prolonged therapeutic time window or use of drugs with broad pan-suppression of inflammatory responses can lead to unexpected or deleterious effects. An example is the detrimental effect observed with chronic administration of ibuprofen in a rat fluid percussion injury model [57].

A number of lines of evidence indicate that brief, selective suppression of the glial activation response to neurologic insults may improve outcomes without compromising the contribution of activated glia to the mechanisms involved in recovery or causing immunosuppression in peripheral tissues [61]. Our data are consistent with this, and support the hypothesis that early interventions to modulate glial activation using a low drug dose may alter disease progression without compromising glial contributions to the mechanisms of neurologic recovery.

## Conclusion

These data add to the evidence identifying glial activation as a therapeutic target in numerous forms of neurologic disease and support two interrelated hypotheses. First, an injury-initiated, self-propagating cytokine cycle can culminate in neurodegeneration [62,63]. Second, this cycle can be targeted therapeutically to alter progression of CNS disorders [29,30,64,65]. The improvements in long-term functional neurologic outcome following suppression of cytokine upregulation in a clinically relevant therapeutic window indicate that selective targeting of neuroinflammation may lead to novel therapies for the major neurologic morbidities resulting from head injury, and indicate the potential of Mzc as a future therapeutic for TBI.

## Abbreviations

AD: Alzheimer's disease; BBB: Blood brain barrier; DG: Dentate Gyrus; IL-1ra: Interleukin receptor antagonist ; IL-1β: Interleukin-1β; IL-6: Interleukin-6; IP: Intraperitoneal; Mzc: Minozac; PoDG: Polymorph Dentate Gyrus; TBI: Traumatic brain injury; TNFα: Tumor necrosis factor α.

## Competing interests

Northwestern University's technology transfer office has licensed Minozac to industry where it is currently under clinical development.

## Authors' contributions

EL carried out the* in vivo *experiments, immunohistochemical analyses and participated in the design of the study. KSM carried out the biochemical and immunohistochemical analyses and participated in the design of the study. LVE and DMW participated in the design and coordination of the study and assisted with the preparation of the manuscript. MSW directed the study, evaluated the data and prepared the manuscript. All authors read and approved the final manuscript.
